# Is there evidence of a ketogenic effect of coconut oil? Commentary: Effect of the Mediterranean diet supplemented with nicotinamide riboside and pterostilbene and/or coconut oil on anthropometric variables in amyotrophic lateral sclerosis. A pilot study

**DOI:** 10.3389/fnut.2023.1333933

**Published:** 2024-01-08

**Authors:** Jakob Norgren, Ingemar Kåreholt, Shireen Sindi

**Affiliations:** ^1^Division of Clinical Geriatrics, Center for Alzheimer Research, Department of Neurobiology, Care Sciences and Society (NVS), Karolinska Institutet, Stockholm, Sweden; ^2^Stockholms Sjukhem, Research and Development Unit, Stockholm, Sweden; ^3^Institute of Gerontology, School of Health and Welfare, Jönköping University, Jönköping, Sweden; ^4^Aging Research Center, Department of Neurobiology, Care Sciences and Society (NVS), Karolinska Institutet and Stockholm University, Stockholm, Sweden; ^5^Neuroepidemiology and Ageing Research Unit, School of Public Health, Imperial College London, London, United Kingdom

**Keywords:** ketosis, ketogenic diet, non-ketogenic, coconut oil, lauric acid, caprylic acid, medium-chain triglycerides, diet terminology

We read with interest the article by Carrera-Juliá et al. (1) including analyses of the effects on anthropometric outcomes of a Mediterranean diet (MeDi)—with a carbohydrate target at 40% of total energy intake (E%)—supplemented with coconut oil. The authors labeled this as a “ketogenic diet” with reference to the content of medium-chain triglycerides (MCTs) in coconut oil. In the present article, we will discuss the terminology of this exposure, which has been used in several publications by the same research group (2–7), because the labeling of the diet as *ketogenic* may be questioned from several perspectives. While many fatty acids, including long-chain (LCFA), may end up in ketogenesis under certain metabolic conditions (8), the focus here is on which specific fatty acids have been demonstrated to substantially increase ketone concentrations, i.e., to the range of nutritional ketosis (9), even in the absence of carbohydrate restriction.

1. To our surprise, a publication of ours (10) was cited to support the claim that “nutritional supplementation with coconut oil could be a good way to promote the synthesis of ketone bodies,” when the conclusion of our study was, in fact, the opposite. Circulating concentrations of the ketone body β-hydroxybutyrate (BHB) were not higher after 30 g intake of coconut oil compared to sunflower oil (which was used as control, not including any MCTs), and intake of coconut oil in combination with carbohydrates did not raise BHB. While mean venous BHB was close to 0.4 mmol/L after the intake of coconut oil *within a 16-h window without carbohydrate intake*, the same was true for sunflower oil, suggesting that the effect was driven by the absence of carbohydrates rather than specific properties of the coconut or sunflower oils.

2. Although both caproic (C6, sparingly consumed), caprylic (C8), capric (C10), and lauric (C12) acid are referred to as medium-chain fatty acids (MCFAs) in the literature, studies on “MCT-oils” typically included triglycerides containing only C8/C10 (11), while the generalizability of their ketogenic properties to C12 may have been unclear. According to several publications since 2017, it now appears that only C8 exhibits a substantial ketogenic effect, but not C10 and C12 (10, 12–14). While C12 constitutes approximately half the fatty acid content of coconut oil, C8 constitutes only 7% (11) (Figure 1)—meaning that even at a daily dose of 60 g [as applied in the study by Carrera-Juliá et al. (1)], coconut oil may not provide substantial ketosis since the C8 content will only be ≈4 g. Findings that C12 was ketogenic in astrocytic cell lines (15) might support speculations on local brain ketogenesis, but as discussed by those authors, “future studies are required to elucidate whether coconut oil intake actually increases the local ketone body production in the brain *in vivo* despite lower hepatic ketogenesis.” We are not aware that such results have been reported anywhere.

**Figure 1:**
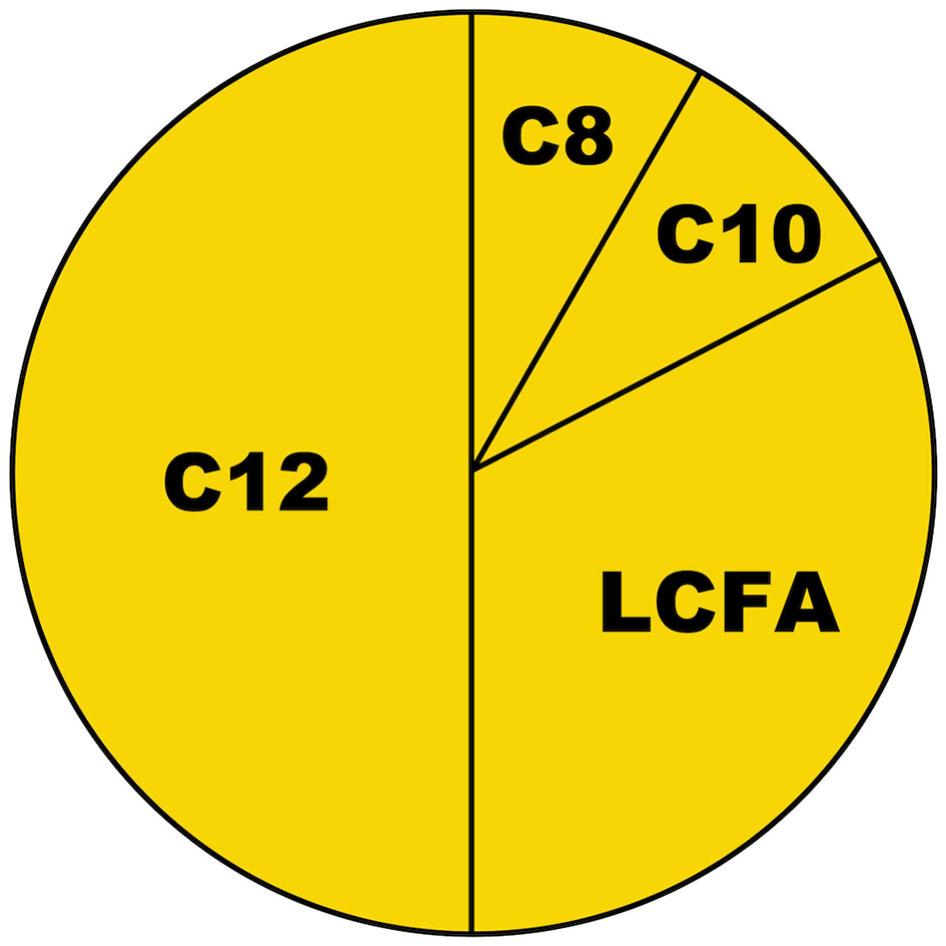
Approximate fatty acid content of coconut oil. C8, C10, and C12 are defined as medium-chain fatty acids (MCFAs) (11). C8, caprylic acid; C10, capric acid; C12, lauric acid; LCFAs, long-chain fatty acids.

3. The suggested BHB range for nutritional ketosis is approximately 0.5–3.0 mmol/L, with possible adjustment depending on whether the measurement is performed in capillary or venous blood (9). Since the study by Carrera-Juliá et al. (1) applied five meals per day with a carbohydrate target of 40 E% for the “ketogenic” MeDi, BHB may not be expected to exceed 0.1 mmol/L (16). A ketogenic diet typically has a carbohydrate limit of 10 E% or even lower, although 20 E% may be allowed in an MCT-enriched ketogenic diet for pediatric epilepsy—where C8 is the main constituent (17).

Similar concerns as ours have been raised regarding another study co-authored by the first and last authors of the current article (3). We stand behind the points discussed in that letter to the editor by Klement (18), and our concerns remain even after reading the authors' response (19). Even though these authors admitted that the label *ketogenic* was not appropriate, previous (5) and subsequent (1, 2, 4, 6, 7) publications from the same research group attributed elevated ketone concentrations as a mechanism of their diet intervention—even though this is unlikely (due to high carbohydrate content and low C8 content). Empirical evidence from their previous articles clearly indicates the absence of ketosis on the “ketogenic” MeDi: Although mean fasting BHB increased significantly from 0.06 to 0.10 mmol/L from pre- to post-intervention (2), this comparison is based on numbers that are below the declared measurement range of the reagent (0.100–5.75 mmol/L; https://www.randox.com/tag/d-3-hydroxybutyrate, assessed 2023-09-28). Moreover, at concentrations below 0.2 mmol/L, BHB may not be a reliable proxy for total ketones since acetoacetate might be the predominant ketone body (9).

From the perspective of the cognitive health field, it is worth noting that one of the aforementioned studies, which applied MeDi—with a 55 E% target for carbohydrates—supplemented with 40 g coconut oil in patients with Alzheimer's disease (5), has been incorporated in several reviews on the impact of ketogenic interventions on cognitive health (20–22), despite unlikely being a ketogenic intervention. We encourage further studies on the potential health effects of diets supplemented with coconut oil but would interpret any such effect as most likely attributable to other mechanisms than ketosis. To exemplify a (probably) non-ketogenic pathway that might promote brain health, C10 and C12 increased the degradation of the amyloid β-protein (23), which may provide a rationale for research on the potential of coconut oil for the prevention of Alzheimer's disease. Further rationales have been reviewed by Fernando et al. (24). The effects of a diet labeled a *modified Mediterranean-ketogenic diet*—with a carbohydrate target of 5–10 E%—have been studied in mild cognitive impairment with promising results (25). However, even in studies on strict carbohydrate restriction where substantial ketosis is confirmed, there may be ambiguity on how important ketosis is relative to other pathways for driving potential effects. Outcomes related to cognitive health (26) (and other health outcomes) may be affected by changes in the carbohydrate/fat ratio even in the non-ketogenic range—leaving a possibility that ketosis is primarily a marker for macronutritional changes and not necessarily the predominant causal mediator. We recently showed that cognitive performance exemplifies a health outcome where the impact of macronutritional composition (in the absence of ketosis) might be substantial in certain subgroups (27). In the current article on anthropometric variables (1), the carbohydrate target differed between MeDi *with* (40 E%) and *without* (51 E%) supplementation with coconut oil, which may provide one alternative explanation for any differences in outcomes.

During the review process of this commentary, we became aware of a recent publication by Fernando et al. (28)—studying the effects of coconut oil supplementation in combination with ≈50 E% carbohydrate intake on cognition in persons with Alzheimer's disease—which calls for additional comments since coconut oil is referred to as a “ketogenic agent,” analogously with the study by Carrera-Juliá et al. (1). No measured ketone concentrations were reported in their study, and after reading the references used to support that MCFAs are ketogenic (29–32), we failed to identify any empirical evidence on the potential ketogenic effect of coconut oil (or C12). In fact, one of those references (31) incorrectly states that “…the major fatty acid in coconut oil being caprylic acid (C8),” and another (30) uses the term MCT with reference to an oil with only 2% C12 but 65–75% C8. The third reference does not define MCFA/MCT (29) and the fourth refers to the MCT trioctanoin (C8) (32). While the results are interesting, our interpretation would be that the study by Fernando et al. (28) did not compare differing ketone concentrations but other factors; one such factor could be macronutritional changes, as discussed by the authors.

To our understanding, inappropriate generalization of empirical results mainly driven by C8 alone to the whole category C6–C12 has been independently performed multiple times in the literature—potentially giving rise to misunderstandings regarding the properties of coconut oil. It might have its origin in the fact that C6–C12 may indeed utilize two “metabolic shortcuts,” as reviewed by Dayrit (11): 1. Uptake from the intestines to the liver via the portal vein; and 2. Passive diffusion into mitochondria without the need for carnitine assistance. Those properties may have been assumed to be sufficient for rapid ketone production, but possibly additional properties, e.g., at the stage of beta-oxidation, are necessary and distinct for C8. Such differences between C8 and C10 have been examined by Sonnay et al. (33), and Christensen et al. showed that C12 may be elongated to LCFA in the carbohydrate-refed state (34). Since MCT inconsistently refers to either C8, C8–C10, or C6–C12, it may be essential to always specify the definition of MCFA/MCT and check references for such definitions when interpreting the literature. Even when we followed the reference chains in a review on the topic (35)—including the statement “Increased ketone levels, obtained through a balanced healthy diet containing ketone precursors such as coconut oil and MCT”—we did not identify empirical evidence targeting coconut oil or C12. Furthermore, a more recent related review (36) states that “dietary medium-chain triglycerides (MCTs) are metabolized into MCFAs (6 to 12 carbons in length) that are then preferentially metabolized into ketone bodies,” while their reference (37) targets a C8-supplement.

Our previous failure to identify relevant evidence on coconut oil or C12 provided the rationale for performing our randomized controlled trial in older adults (10), which corroborated findings in younger adults that had just been published by Vandenberghe et al. (14), indicating that C8 but not coconut oil was substantially ketogenic. Similar results were later reported by Baumeister et al. (5), adding analyses on the additional impact of caffeine.

Within this commentary, we have not made any evaluation of any of the discussed articles beyond examining the references used to claim that coconut oil would be ketogenic. We have shown that some concerns regarding the article by Carrera-Juliá et al. (1) extend to multiple publications in the field. It may not be excluded that we have missed identifying relevant evidence, and we welcome any suggestion on a study reporting ketone concentrations indicating ketosis after the intake of coconut oil or C12 in the absence of carbohydrate restriction or additional C8 intake. Until such evidence has been presented, it may not be appropriate to define coconut oil supplementation as a ketogenic intervention. In conclusion, regardless of the condition of interest, the term *ketogenic* may be interpreted with caution in the literature—not necessarily pointing toward the predominant mediator at work.

## Author contributions

JN: Conceptualization, Writing – original draft. IK: Writing – review & editing. SS: Writing – review & editing.

## References

[1] Carrera-Juliá S Estrela JM Zacarés M Navarro MÁ Vega-Bello MJ de la Rubia Ortí JE. Effect of the Mediterranean diet supplemented with nicotinamide riboside and pterostilbene and/or coconut oil on anthropometric variables in amyotrophic lateral sclerosis. A pilot study. Front Nutr. (2023) 10:1232184. 10.3389/fnut.2023.1232184 37810917 PMC10556480

[2] Benlloch M Cuerda Ballester M Drehmer E Platero JL Carrera-Juliá S López-Rodríguez MM. Possible reduction of cardiac risk after supplementation with epigallocatechin gallate and increase of ketone bodies in the blood in patients with multiple sclerosis. A pilot study. Nutrients. (2020) 12:3792. 10.3390/nu12123792 33322022 PMC7763038

[3] Benlloch M López-Rodríguez MM Cuerda-Ballester M Drehmer E Carrera S Ceron JJ. Satiating effect of a ketogenic diet and its impact on muscle improvement and oxidation state in multiple sclerosis patients. Nutrients. (2019) 11:1156. 10.3390/nu11051156 31126118 PMC6566517

[4] Cuerda-Ballester M Proaño B Alarcón-Jimenez J de Bernardo N Villaron-Casales C Lajara Romance JM. Improvements in gait and balance in patients with multiple sclerosis after treatment with coconut oil and epigallocatechin gallate. A pilot study. Food Funct. (2023) 14:1062–71. 10.1039/D2FO02207A 36594273

[5] Orti JED Garcia-Pardo MP Drehmer E Cantus DS Rochina MJ Calpe MAA. Improvement of main cognitive functions in patients with Alzheimer's disease after treatment with coconut oil enriched mediterranean diet: a pilot study. J Alzheimers Dis. (2018) 65:577–87. 10.3233/JAD-180184 30056419

[6] de la Rubia Orti JE Platero JL Yang IH Ceron JJ Tvarijonaviciute A Sabater PS. Possible role of butyrylcholinesterase in fat loss and decreases in inflammatory levels in patients with multiple sclerosis after treatment with epigallocatechin gallate and coconut oil: a pilot study. Nutrients. (2021) 13:3230. 10.3390/nu13093230 34579104 PMC8465111

[7] Platero JL Cuerda-Ballester M Sancho-Cantus D Benlloch M Ceron JJ Peres Rubio C. The impact of epigallocatechin gallate and coconut oil treatment on cortisol activity and depression in multiple sclerosis patients. Life (Basel). (2021) 11:353. 10.3390/life11040353 33920655 PMC8073508

[8] Cunnane SC. Ketones, omega-3 fatty acids and the Yin-Yang balance in the brain: insights from infant development and Alzheimer's disease, and implications for human brain evolution. Ocl-Oilseeds Fats Crops Lipids. (2018) 25:20. 10.1051/ocl/2018020

[9] Norgren J Sindi S Sandebring-Matton A Kareholt I Akenine U Nordin K. Capillary blood tests may overestimate ketosis: triangulation between three different measures of beta-hydroxybutyrate. Am J Physiol Endocrinol Metabol. (2020) 318:E184–E8. 10.1152/ajpendo.00454.2019 31821040

[10] Norgren J Sindi S Sandebring-Matton A Kareholt I Daniilidou M Akenine U. Ketosis after intake of coconut oil and caprylic acid-with and without glucose: a cross-over study in healthy older adults. Front Nutr. (2020) 7:40. 10.3389/fnut.2020.00040 32351966 PMC7175812

[11] Dayrit FM. The properties of lauric acid and their significance in coconut oil. J Am Oil Chem Soc. (2014) 92:1–15. 10.1007/s11746-014-2562-7

[12] Baumeister A Gardemann J Fobker M Spiegler V Fischer T. Short-term influence of caffeine and medium-chain triglycerides on ketogenesis: a controlled double-blind intervention study. J Nutr Metab. (2021) 2021:1861567. 10.1155/2021/1861567 34221499 PMC8221889

[13] St-Pierre V Vandenberghe C Lowry CM Fortier M Castellano CA Wagner R. Plasma ketone and medium chain fatty acid response in humans consuming different medium chain triglycerides during a metabolic study day. Front Nutr. (2019) 6:46. 10.3389/fnut.2019.00046 31058159 PMC6481320

[14] Vandenberghe C St-Pierre V Pierotti T Fortier M Castellano CA Cunnane SC. Tricaprylin alone increases plasma ketone response more than coconut oil or other medium-chain triglycerides: an acute crossover study in healthy adults. Curr Dev Nutr. (2017) 1:257. 10.3945/cdn.116.000257 29955698 PMC5998344

[15] Nonaka Y Takagi T Inai M Nishimura S Urashima S Honda K. Lauric Acid Stimulates Ketone Body Production in the KT-5 Astrocyte Cell Line. J Oleo Sci. (2016) 65:693–9. 10.5650/jos.ess16069 27430387

[16] Cahill GF. Fuel metabolism in starvation. Annu Rev Nutr. (2006) 26:1–22. 10.1146/annurev.nutr.26.061505.111258 16848698

[17] Liu YM Wang HS. Medium-chain triglyceride ketogenic diet, an effective treatment for drug-resistant epilepsy and a comparison with other ketogenic diets. Biomed J. (2013) 36:9–15. 10.4103/2319-4170.107154 23515148

[18] Klement RJ. When is a ketogenic diet ketogenic? Comment on “satiating effect of a ketogenic diet and its impact on muscle improvement and oxidation state in multiple sclerosis patients, Nutrients 2019, 11, 1156”. Nutrients. (2019) 11:1909. 10.3390/nu11081909 31443214 PMC6722583

[19] Benlloch M López-Rodríguez MM Cuerda-Ballester M Drehmer E Carrera S Ceron JJ. Reply to “When is a ketogenic diet ketogenic? Comment on satiating effect of a ketogenic diet and its impact on muscle improvement and oxidation state in multiple sclerosis patients. Nutrients. (2019) 11:1919. 10.3390/nu11081919 31126118 PMC6566517

[20] Bohnen JLB Albin RL Bohnen NI. Ketogenic interventions in mild cognitive impairment, Alzheimer's disease, and Parkinson's disease: a systematic review and critical appraisal. Front Neurol. (2023) 14:1123290. 10.3389/fneur.2023.1123290 36846143 PMC9947355

[21] Devranis P Vassilopoulou E Tsironis V Sotiriadis PM Chourdakis M Aivaliotis M. Mediterranean Diet, ketogenic diet or MIND diet for aging populations with cognitive decline: a systematic review. Life (Basel). (2023) 13:173. 10.3390/life13010173 36676122 PMC9866105

[22] Tabaie EA Reddy AJ Brahmbhatt H. A narrative review on the effects of a ketogenic diet on patients with Alzheimer's disease. AIMS Public Health. (2022) 9:185–93. 10.3934/publichealth.2022014 35071677 PMC8755961

[23] Mett J Lauer AA Janitschke D Griebsch LV Theiss EL Grimm HS. Medium-chain length fatty acids enhance abeta degradation by affecting insulin-degrading enzyme. Cells. (2021) 10:2941. 10.3390/cells10112941 34831163 PMC8616162

[24] Fernando WM Martins IJ Goozee KG Brennan CS Jayasena V Martins RN. The role of dietary coconut for the prevention and treatment of Alzheimer's disease: potential mechanisms of action. Br J Nutr. (2015) 114:1–14. 10.1017/S0007114515001452 25997382

[25] Neth BJ Mintz A Whitlow C Jung Y Solingapuram Sai K Register TC. Modified ketogenic diet is associated with improved cerebrospinal fluid biomarker profile, cerebral perfusion, and cerebral ketone body uptake in older adults at risk for Alzheimer's disease: a pilot study. Neurobiol Aging. (2020) 86:54–63. 10.1016/j.neurobiolaging.2019.09.015 31757576 PMC7266642

[26] Norgren J Sindi S Sandebring-Matton A Ngandu T Kivipelto M Kåreholt I. The dietary carbohydrate/fat-ratio and cognitive performance: panel analyses in older adults at risk for dementia. Curr Dev Nutr. (2023) 7:100096. 10.1016/j.cdnut.2023.100096 37275847 PMC10236460

[27] Norgren J Sindi S Matton A Kivipelto M Kåreholt I. APOE-genotype and insulin modulate estimated effect of dietary macronutrients on cognitive performance: panel analyses in non-diabetic older adults at risk for dementia. J Nutr. (2023) 153:3506–20. 10.1016/j.tjnut.2023.09.016 37778510

[28] Fernando MG Silva R Fernando W de Silva HA Wickremasinghe AR Dissanayake AS. Effect of virgin coconut oil supplementation on cognition of individuals with mild-to-moderate Alzheimer's disease in Sri Lanka (VCO-AD Study): a randomized placebo-controlled trial. J Alzheimer's Dis. (2023) 96:670. 10.3233/JAD-230670 37980665

[29] Aoyama T Nosaka N Kasai M. Research on the nutritional characteristics of medium-chain fatty acids. J Med Investig. (2007) 54:385–8. 10.2152/jmi.54.385 17878693

[30] Babayan VK. Medium chain triglycerides and structured lipids. Lipids. (1987) 22:417–20. 10.1007/BF02537271 3112486

[31] Ruppin DC Middleton WR. Clinical use of medium chain triglycerides. Drugs. (1980) 20:216–24. 10.2165/00003495-198020030-00005 7428670

[32] Valdivieso V. Absorption of medium-chain triglycerides in animals with pancreatic atrophy. Am J Dig Dis. (1972) 17:129–37. 10.1007/BF02232732 5013492

[33] Sonnay S Chakrabarti A Thevenet J Wiederkehr A Christinat N Masoodi M. Differential metabolism of medium-chain fatty acids in differentiated human-induced pluripotent stem cell-derived astrocytes. Front Physiol. (2019) 10:657. 10.3389/fphys.2019.00657 31214043 PMC6558201

[34] Christensen E Hagve TA Gronn M Christophersen BO. Beta-oxidation of medium chain (C8-C14) fatty acids studied in isolated liver cells. Biochim Biophys Acta. (1989) 1004:187–95. 10.1016/0005-2760(89)90267-1 2752017

[35] Chatterjee P Fernando M Fernando B Dias CB Shah T Silva R. Potential of coconut oil and medium chain triglycerides in the prevention and treatment of Alzheimer's disease. Mech Ageing Dev. (2020) 186:111209. 10.1016/j.mad.2020.111209 31953123

[36] Castro CB Dias CB Hillebrandt H Sohrabi HR Chatterjee P Shah TM. Medium-chain fatty acids for the prevention or treatment of Alzheimer's disease: a systematic review and meta-analysis. Nutr Rev. (2023) 81:1144–62. 10.1093/nutrit/nuac104 36633304

[37] Henderson ST Vogel JL Barr LJ Garvin F Jones JJ Costantini LC. Study of the ketogenic agent AC-1202 in mild to moderate Alzheimer's disease: a randomized, double-blind, placebo-controlled, multicenter trial. Nutr Metab (Lond). (2009) 6:31. 10.1186/1743-7075-6-31 19664276 PMC2731764

